# Synergistic effect of gefitinib and rofecoxib in mesothelioma cells

**DOI:** 10.1186/1476-4598-9-27

**Published:** 2010-02-02

**Authors:** Daniela Stoppoloni, Claudia Canino, Irene Cardillo, Alessandra Verdina, Alfonso Baldi, Ada Sacchi, Rossella Galati

**Affiliations:** 1 Department for the Development of Therapeutic Programs, Laboratory D, Centro Ricerca Sperimentale, Regina Elena Cancer Institute, Via delle Messi D'Oro 156, 00158 Rome, Italy; 2 Department of Biochemistry and Biophysic "F Cedrangolo", Section of Anatomic Pathology, Second University of Naples, Via L Amari 5, 80138 Naples, Italy

## Abstract

**Background:**

Malignant mesothelioma (MM) is an aggressive tumor that is resistant to conventional modes of treatment with chemotherapy, surgery or radiation. Research into the molecular pathways involved in the development of MM should yield information that will guide therapeutic decisions. Epidermal growth factor receptor (EGFR) and cyclooxygenase-2 (COX-2) are involved in the carcinogenesis of MM. Combination of COX-2 and EGFR inhibitors, therefore, could be an effective strategy for reducing cell growth in those lines expressing the two molecular markers.

**Results:**

In order to verify the effect of COX-2 and EGFR inhibitors, five MM cell lines NCI-2452, MPP89, Ist-Mes-1, Ist-Mes-2 and MSTO-211 were characterized for COX-2 and EGFR and then treated with respective inhibitors (rofecoxib and gefitinib) alone and in combination. Only MPP89, Ist-Mes-1 and Ist-Mes-2 were sensitive to rofecoxib and showed growth-inhibition upon gefitinib treatment. The combination of two drugs demonstrated synergistic effects on cell killing only in Ist-Mes-2, the cell line that was more sensitive to gefitinib and rofecoxib alone. Down-regulation of COX-2, EGFR, p-EGFR and up-regulation of p21 and p27 were found in Ist-Mes-2, after treatment with single agents and in combination. In contrast, association of two drugs resulted in antagonistic effect in Ist-Mes-1 and MPP89. In these cell lines after rofecoxib exposition, only an evident reduction of p-AKT was observed. No change in p-AKT in Ist-Mes-1 and MPP89 was observed after treatment with gefitinib alone and in combination with rofecoxib.

**Conclusions:**

Gefitinib and rofecoxib exert cell type-specific effects that vary between different MM cells. Total EGFR expression and downstream signalling does not correlate with gefitinib sensitivity. These data suggest that the effect of gefitinib can be potentiated by rofecoxib in MM cell lines where AKT is not activated.

## Background

Malignant mesothelioma (MM) is a fatal malignancy with an estimated incidence of 3,000 cases per year in the United States. In the next 30 years in Western Europe, 250,000 deaths are envisaged [1]. There is no standard of care for MM, and current treatments, ranging from aggressive surgical treatment to chemotherapy, fail to improve the disease prognosis [2]. MM occurs in a context of asbestos exposure and chronic inflammation, such as would be expected to enhance the expression of inducible enzymes which cyclooxygenase (COX). Two COX isoforms have been identified as COX-1 and COX-2 [3]. COX-1 is expressed constitutively in several cell types of normal mammalian tissues, where it is involved in the maintenance of tissue homeostasis. In contrast, COX-2 is an inducible enzyme responsible for prostaglandin-E2 (PGE2) production at sites of inflammation [3]. Cyclooxygenase activity occurs in cultured human MM cells and COX-2 is induced by inflammatory cytokines [4]. Nevertheless, COX-2 expression is a strong prognostic factor in human MM, which contributes independently of the other clinical and histopathological factors in determining a short survival [5].

Several studies have shown that non-steroidal anti-inflammatory drugs (NSAID) are able to prevent the development of various human cancers, including MM [6-8], even if the exact molecular mechanisms in chemoprevention of NSAID are not clearly understood. There is good correlation between high levels of COX-2 and tumour cell sensitivity to NSAIDs [9]. As a result, COX-2 has become a natural target for anti-cancer agents [10] and selective COX-2 inhibitors, such as celecoxib and rofecoxib, have been considered for therapy [11,12]. The induction of COX-2 and up-regulation of the prostaglandin cascade play a significant role in carcinogenesis by promoting cell division [13], induction of vascular endothelial growth factor and stimulation of an antiapoptotic pathway [14]. In turn, COX 2 may be additionally up-regulated as a positive feedback mechanism by EGFR pathway [15]. EGFR, a receptor tyrosine kinase , is over-expressed in a wide variety of epithelial malignancies including MM [16]. It is known that 68% of MM specimens show EGFR expression [17]. In rat pleural MM cells, the phosphorylation of EGFR appears to correlate with the carcinogenicity of the asbestos fibers, with a greater degree of phosphorylation observed after treatment with fibrous preparations [18]. Asbestos fibers also induced the phosphorylation of mitogen-activated protein kinase and extracellular signal-regulated kinase (ERK) 1 and 2 [19]. EGFR appears to be involved in the constitutive activation of the phosphoinositide-3-kinase (PI3/AKT) signalling pathway in MM cell lines and other solid tumors as well as in their resistance to treatment, such as radiation and chemiotherapy [20]. Phosphorylated AKT conveys downstream signals, promoting cellular proliferation and survival [21].

Several strategies have been developed for targeting EGFR, including low molecular weight tyrosine kinase inhibitors [22]. Gefitinib (Iressa, ZD-1839) acts as a competitive inhibitor of ATP for binding to the EGFR tyrosine kinase pocket [23] and inducing the formation of inactive EGFR dimers and homodimers [24]. EGFR inhibitors have been shown to be effective in preclinical studies and animal models and are in the final stages of clinical trials [25]. Besides, the interaction between the EGFR and COX-2 pathways [26,15] could suggest that targeting both EGFR and COX-2 may be an effective approach to modulate both pathways and their downstream signalling, which may result in an increased therapeutic response in MM.

The combination of COX-2 and EGFR inhibitor was shown to have a synergistic effect in cancer treatments [27]. Combined treatment with a COX-2 inhibitor and an EGFR-TKI has been shown to inhibit the EGFR-mediated pathways, including ERK and AKT [28]. Based on the relevance of the COX-2 and EGFR pathways in MM [4,5,17,18] and the overlap between the two pathways [15], we performed studies to characterize five MM cell lines for COX and EGFR signalling and to analyze their response to COX-2 and EGFR inhibitors as single agent or in combination.

## Methods

### Cell Lines

The human MM cell lines MSTO-211H and NCI-H2452 were obtained from the American Type Culture Collection (Rockville, MD). Cells were cultured as monolayers in flasks using ATCC complete growth medium in a humidified atmosphere containing 5% CO_2 _at 37°C. The human MM cell lines Ist-Mes-1, Ist-Mes-2 and MPP89 were obtained from the Genova Institute Culture Collection. Ist-Mes-1 and Ist-Mes-2 were cultured in Dulbecco's modified eagle medium (DMEM) with piruvate, supplemented with 10% FBS, glutamine (2 mM), 1% non essential aminoacids and antibiotics (0.02 IU.mL/1 penicillin and 0.02 mg.mL/1 streptomycin) while the established MM cell line, MPP89, was maintained in Ham's F10 with 15% FBS, and supplemented with glutamine (2 mM) and antibiotics (0.02 IU.mL/1 penicillin and 0.02 mg.mL/1 streptomycin) in a humidified atmosphere containing 5% CO_2 _at 37°C.

### Drugs

Gefitinib and rofecoxib (Sequoia Research Product, UK) stock solutions were prepared in DMSO and stored at -20°C. The drugs were diluted in fresh media before each experiment. EGF was purchased from Biosource International Inc. (Camarillo, CA), dissolved in distilled H2O and stored at -70°C before use.

### Protein Extraction and Western Blot Analysis

MM cells were used to determine the baseline expression of the COXs, EGFR and EGFR phosphorylation. Ist-Mes-1, Ist-Mes-2 and MPP89 were treated with EGF to increase the level of EGFR phosphorylation. Cells were seeded in full culture media for 24 h before 100 ng/ml of EGF was added for 15 and 30 min. MM cells were lysed in ice-cold lysis buffer (20 mM Tris (pH 8.0), 150 mM NaCl, 10% glycerol, 1% NP40, and 0.42% NaF) containing proteinase and phosphatase inhibitors (Pierce Biotechnology) and separated on SDS-PAGE. The separated proteins were transferred onto nitrocellulose membranes, blocked with 5% milk, and incubated overnight at 4°C with antibodies against the phosphorylated proteins. After 1 h incubation with the horseradish peroxidase-conjugated secondary antibody, the phosphorylated proteins were revealed by ECL Western blotting detection reagents (Amersham Pharmacia; Uppsala, Sweden) according to the manufacturer's instructions.

Membranes were stripped by incubation in 1 M Tris-HCl (pH 6.8), 10% SDS, and 10 mM dithiotreitol for 30 min at 55°C, and re-probed with antibodies of interest. Goat anti mouse or rabbit IgG horseradish peroxidase-conjugated secondary antibodies (1:3,000) (Bio-Rad Laboratories; Hercules, CA, USA) was used. The blots were then reacted with ECL Western blotting detection reagents and intensity assessed by densitometric analysis of digitalized autoradiographic images using Scion Image software. Actin was used as a loading control. The experiments were performed in triplicate. Proteins were probed with antibodies against COX-2 (monoclonal antibody Cayman Chemical (1:500) , EGFR-1005 (1:1,000) and phospho-specific EGFR p-Tyr-PY20 (1:100), p27 and p21 (1:250) (S. Cruz Biotechnology, Santa Cruz, CA, USA), AKT (1:1,000), pAKT (1:1,000), ERK1/2 (1:1,000) and pERK1/2 (1:1,000) (Cell Signaling Technology) and monoclonal anti actin (1:10,000) (Sigma, Saint Louis Missouri, USA).

### RNA isolation and RT-PCR

Total RNA was prepared from cultured MSTO-211H, NCI-H2452, Ist-Mes-1, Ist-Mes-2 and MPP89 using TRIzol Reagent (Invitrogen Life Technologies, Paisley, UK) according to the manufacturer's protocols. Reverse transcription of RNA, for first-strand cDNA synthesis, was performed using 4 μg total RNA and 0.5 μg oligo (dT) 12-18 primer (Invitrogen Life Technologies, Paisley, UK), 10 mM dNTP mix in a final volume of 12 μl. The reaction was incubated at 70°C for 10 min and immediately chilled on ice. Primer extension was then performed for 10 mins at room temperature and 42°C for 2 mins following addition of First -Strand Buffer, 10 mM dithiothreitol, and 40U RNase OUT Recombinant Ribonuclease Inhibitor (Invitrogen Life Technologies, Paisley, UK) in a final volume of 19 μl. 1 μl (200U) SuperScript II Reverse Transcriptase was then added (Invitrogen Life Technologies, Paisley, UK) and incubated at 42°C for 50 min. The reaction was inactivated by heating at 70°C for10 mins. cDNA was stored at -20°C.

Quantitative PCR was conducted in a volume of 25 μl containing 40 ng cDNA (1/100 dilution of reverse transcriptase mixture), 1.25 μl of primer (COX-2 or EGFR) and 12.5 μl TaqMan Universal PCR Master Mix (Applied Biosystems, Foster City, CA, USA) in the following sequence: 2 mins at 50°C and denaturation for 10 mins at 95°C followed by 40 cycles of the amplification step at 95° for 15 secs (denaturation), and then at 60°C for 60 secs (annealing/extension) in 96-well plates using the ABI PRISM 7000 sequence Detection System (Applied Biosystems, Foster City, CA, USA). Quantitative PCR for the endogenous control glyceraldehyde-3-phosphate dehydrogenase (GAPDH) was carried out under the same conditions, using a GAPDH Assay on Demand (Applied Biosystems, Foster City, CA, USA). A standard curve for COX-2 and EGFR genes was constructed using serial dilutions (200-40-8-1.6 ng) from a pool of cDNAs from MSTO, NCI, Ist-Mes-1, Ist-Mes-2 and MPP89 cells. Results were analyzed using the Applied Biosystems analysis software and expression levels calculated from a linear regression of the standard curve. Results are given as gene expression vs GAPDH expression (COX-2 or EGFR relative expression) to correct for differences in the quantity of cDNA used in the PCR reaction. All quantitative PCR reactions for each sample were performed in triplicate.

### In Vitro Cytotoxicity Assays

The in vitro drug sensitivity was assessed by Cell Proliferation kit (XTT) (Roche Molecular Biochemicals, Indianapolis, IN), using the manufacturer's instructions. The assay is based on the cleavage of the yellow tetrazolium salt XTT to form an orange formazan dye by metabolic active cells. This conversion only occurs in viable cells. The formazan dye formed is soluble in aqueous solutions and is directly quantified using a scanning multiwell spectrophotometer at 492 nm with a reference wavelength at 650 nm. Cells were seeded at 2,500-20,000 cells/well in 96-well flat-bottomed plate (Corning Inc., Corning, NY) to allow for an exponential growth for the 3 days of the assay to give an absorbance of 1.0-2.2. The optimum number of cells required to reach an absorbance between 1.0 and 2.2 was determined for each cell line (data not shown). In a typical experiment, cells were trypsinized, seeded in 96-well plates, and allowed to recover for 24 h before the addition of gefitinib or rofecoxib or gefitinib and rofecoxib together. Drug concentrations ranged from 6.25 μM to 50 μM for gefitinib, 4 μM to 36 μM for rofecoxib. The concentration of drugs required to obtain a 25% inhibition (IC_25_) of proliferation of Ist-Mes-2, was used to test the effectiveness of the rofecoxib and gefitinib association in each cell line. To 25 μM gefitinib (Ist-Mes-2 IC_25_) were added 4, 12 and 36 μM of rofecoxib and to 12 μM rofecoxib was added 12, 5 μM gefitinib. All experimental points were quantified fivefold. Every single point was compared to their respective control with the same amount of DMSO. All experiments were repeated three times. The assay was developed after 48 h incubation and absorbance was then measured. The cytotoxic effect obtained with the gefitinib and rofecoxib combinations was analysed according to the Chou and Talalay method [29]. Combination index (CI) values above 1.1 indicate antagonistic, 0.9 to 1.1 additive, 0.7 to 0.9 moderately synergistic, 0.3 to 0.7 synergistic, and <0.3 strongly synergistic.

### Drug treatment

The anti-proliferative activity of single drug treatments was assessed in a monolayer culture condition by plating Ist-Mes-1, Ist-Mes-2 and MPP89 cells in T25 flask. After 24 h, DMSO (at the same final concentration of that present in medium with drugs), 50 μM gefitinib or 36 μM rofecoxib were added. The cells were then harvested at 48 h after treatment and analyzed by western blot and RT-PCR to evaluate the effect of the drugs on expression and mRNA levels of EGFR and COX-2. The expression of the cell cycle arrest genes and p-AKT, AKT, p-ERK and ERK was detected by Western blot (as described above) to assess the antiproliferative activity of the two drugs in isolation (25 μM gefitinib or 4 μM rofecoxib) and in combination 25 μM gefitinib+4 μM rofecoxib.

### Treatment of MM cells with Gefitinib and EGF

To determine the effect of gefitinib on the phosphorylation status of EGFR, Ist-Mes-1, Ist-Mes-2 and MPP89, cells were seeded in T25 flask in full culture media for 24 h. 45 mins after the addition of gefitinib, EGF (final concentration 100 ng/ml) was added. DMSO was added to the control medium to give a final concentration that matched DMSO present in medium containing drugs. The cells were harvested 1 h after gefitinib addition, lysed and analyzed by RT-PCR and Western blot as described above. Cells treated with EGF for 15 mins were used to control for EGF-induced phosphorylation.

### Statistical Analysis

Comparisons of treatment outcomes were tested for statistical differences using the Student t-test for paired data. Statistical significance was assumed at a P-value of ≤ 0.05.

## Results

### Effect of rofecoxib on the viability of MM cells

**Figure 1:**
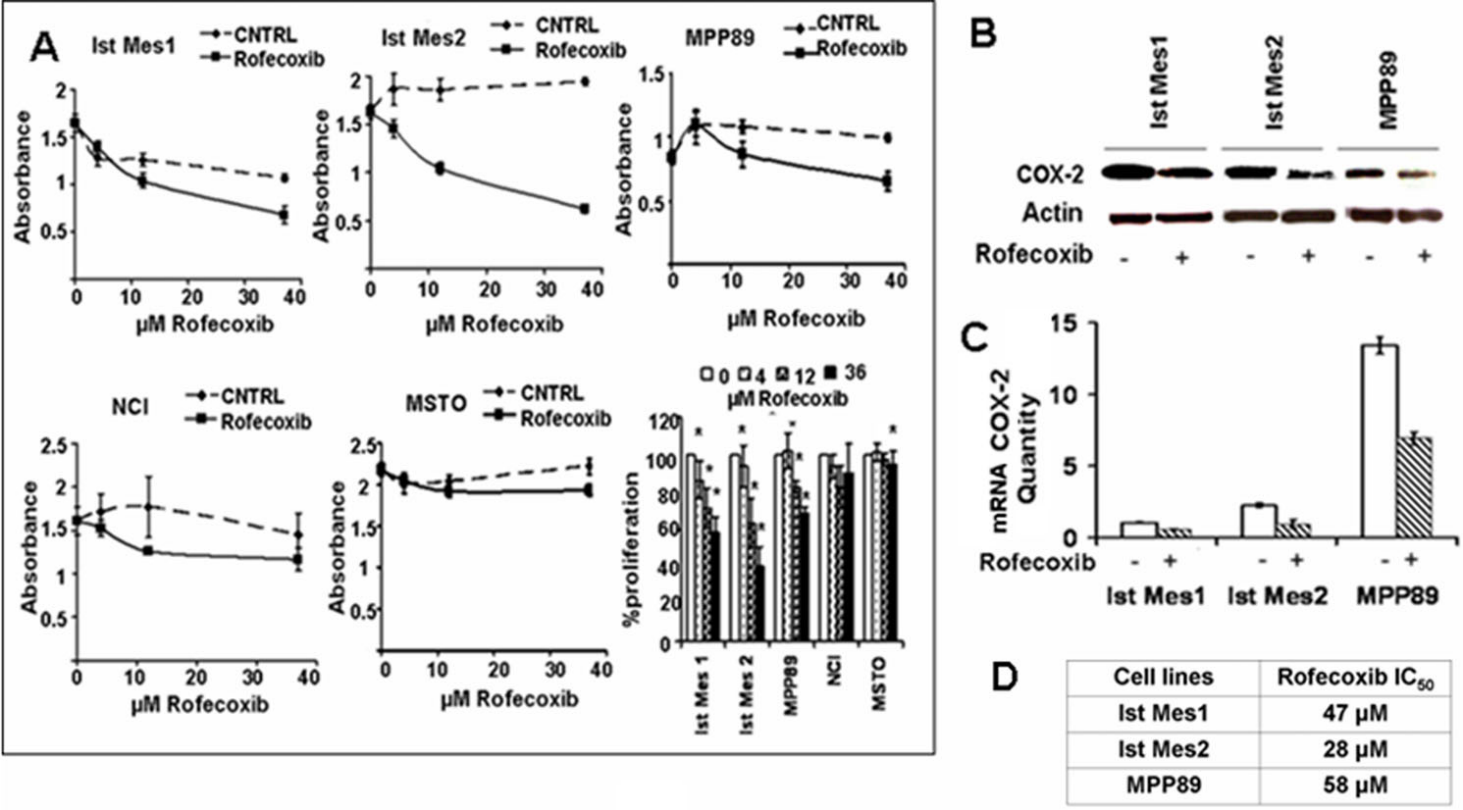
**Curves of cell proliferation and COX-2 modulation after addition of rofecoxib at various concentrations in the MM cell lines**. **A**, the graphs represent cell growth curves of the five cell lines treated with 4, 12, and 36 μM of drug (Rofecoxib) compared to cells treated with DMSO at the same final concentration of that present in medium with drugs (CNTRL) as described in material and methods. The survival of cells was expressed as absorbance (A _492 nm_-A_690 nm_) and % of proliferation (on the right bottom). To evaluate the modulation of rofecoxib on COX-2, Ist-Mes-1, Ist-Mes-2 and MPP89 cell lines were treated with 36 μM rofecoxib for 48 h. The effect of the drug was evaluated by Western blot (**B**) and quantitative PCR (**C**). The table (**D**) displays IC_50 _in the cell lines more sensitive to rofecoxib.

Cell growth of MM cell lines treated with rofecoxib, of doses ranging from 4 to 36 μM, was determined by the cell proliferation kit. Figure 1A shows the effect of rofecoxib on the survival of the five MM cell lines. The largest dose of drug caused a cell proliferation of 68% in MPP89, of 58% in Ist-Mes-1 and 40% in Ist-Mes-2. MSTO-211H and NCI-H2452 treated with 36 μM of rofecoxib had a survival of 97% and 90% respectively, when compared with their controls. The concentration of drug required to obtain a 50% inhibition of proliferation in vitro (IC_50_) was determined only in the cell lines most sensitive to the drugs (Figure 1D). In detail, IC50 was obtained by extrapolation from the cytotoxicity curve. Despite the fact that extrapolation may not be the best method with which to calculate the IC50, it provided us with an indication of the different sensitivity of cell lines. In the same cell lines the effect of rofecoxib on COX-2 was also tested. Ist-Mes-1, Ist-Mes-2 and MPP89 cell lines incubated with 36 μM rofecoxib for 48 h showed a significant decrease in both COX-2 and mRNA levels (Figure 1B and 1C), indicating a specific effect of rofecoxib on COX2.

### EGFR signalling in MM cells

**Figure 2:**
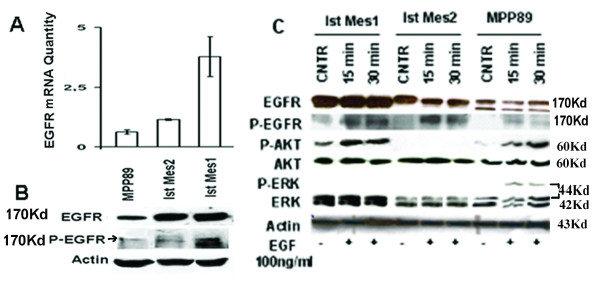
**Characterization of EGFR in the MM cell lines**. Levels of mRNA were revealed by quantitative PCR (**A**), EGFR RNA quantity indicates EGFR gene expression vs GAPDH. The standard deviation expresses the result of three different quantizations and expression of EGFR protein was revealed by quantitative PCR. The western blot (**B**) shows the levels of EGFR and phosphorylated EGFR protein (P-EGFR) in MM cell lines. Cell extracts (100 μg) were probed for phospho-specific EGFR p-Tyr-PY20 and detected with ECL. Blots were stripped and then re-probed for EGFR. Actin was used as loading control. **C**, effect of EGF on phosphorylation of EGFR, AKT and ERK. The cells were cultured in complete medium for 24 and then treated with EGF (100 ng/mL) for 15 and 30 min. Western blot of total lysates (60 μg) indicates that the addition of EGF at a concentration of 100 ng/mL significantly increased EGFR phosphorylation already after 15 minutes. AKT also become phosphorylated in Ist-Mes-1 and MPP89, whereas ERK become phosphorylated in MPP89.

Basal level of EGFR transcript was detected by RT-PCR and Western blot in MPP89, Ist-Mes-2 and Ist-Mes-1 cell lines, at a lower level in MPP89 (Figure 2A and 2B). Different levels of EGFR phosphorylation (P-EGFR) were detected in the cell lines analyzed (Figure 2B). In Ist-Mes-1, Ist-Mes-2 and MPP89 cell lines, the addition of EGF at a concentration of 100 ng/mL significantly increased EGFR phosphorylation after 15 mins, when compared with the control. (Figure 2C). Exogenous EGF trigged a further increase in ERK and AKT phosphorylation in MPP89 and AKT phosphorylation in Ist-Mes-1, indicating that the EGF-EGFR pathway was activated in these cell lines. Interestingly, in Ist-Mes-2 cells, EGF stimulation induced EGFR phosphorylation as expected, but did not induce AKT and ERK phosphorylation, suggesting other possible signalling pathways of EGFR.

### Effect of gefitinib on the viability of MM cells

**Figure 3:**
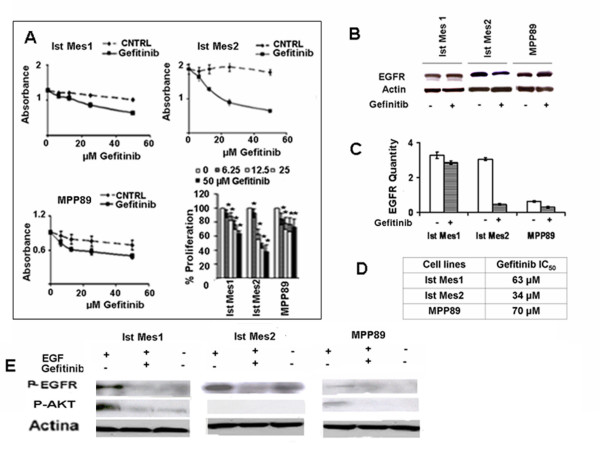
**Curves of cell proliferation and EGFR modulation after addition of gefitinib at various concentrations**. **A**, the graphs represent cell growth curves of the Ist-Mes-1, Ist-Mes-2 and MPP89 cell lines treated with 6.25, 12.5, 25 and 50 μM of drug (Gefitinib) compared to cells treated with DMSO at the same final concentration of that present in medium with drugs (CNTRL) as described in material and methods. The survival of cells was expressed as absorbance (A _492 nm_-A_690 nm_) and % of proliferation (on the right bottom). To evaluate the modulation of gefitinib on EGFR, Ist-Mes-1, Ist-Mes-2 and MPP89 cell lines were treated with 50 μM gefitinib for 48 h. The effect of the drug was evaluated by western blot (**B**) and quantitative PCR (**C**). The table (**D**) displays gefitinib IC_50 _in the cell lines. **E**, effect of gefitinib on EGFR and AKT phosphorylation. Ist-Mes-1, Ist-Mes-2 and MPP89 cells were cultured in complete medium for 24 hours and then treated with gefitinib 50 μM as described in materials and methods. EGFR-phosphorylation (P-EGFR) and AKT-phosphorylation (P-AKT) were analyzed by western blot as described above. Cell treated with EGF were used to control for EGF-induced phosphorylation. DMSO was added to the medium of control to give a final concentration that matched DMSO present in medium containing drugs.

Cell growth of MPP89, Ist-Mes-1, Ist-Mes-2 cells treated with gefitinib at doses ranging from 6.25 to 50 μM, was determined by the cell proliferation kit. In MPP89, Ist-Mes-1 and especially in Ist-Mes-2, a significant growth inhibition by gefitinib was observed (Figure 3A). In figure 3D the IC_50 _demonstrates the greater drug sensitivity in Ist-Mes-2 than in Ist-Mes-1 and MPP89. In Ist-Mes-1 and MPP89 cell lines, upon gefitinib treatment, only a mild decrease in the EGFR mRNA level was observed (Figure 3C). In Ist-Mes-2 cell, gefitinib treatment resulted in a significant decrease in EGFR protein (Figure 3B), as well as mRNA level (Figure 3C). Significant inhibition of phosphorylation of the EGFR was observed with gefitinib (50 μM) in the EGF-treated Ist-Mes-2 (Figure 3E). AKT phosphorylated was not detected in this cell line upon EGF treatment. However, exogenous EGF triggered further increases in EGFR and AKT phosphorylation in the Ist-Mes-1 and MPP89 cells that were reversed by gefitinib (Figure 3E).

### Effect of gefitinib and rofecoxib combination on the viability of MM cells

**Figure 4:**
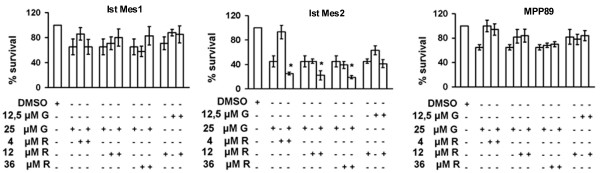
**Curves of cell proliferation after the addition simultaneously of Rofecoxib and Gefitinib**. Ist-Mes-1, Ist-Mes-2 and MPP89 were treated with four different associations: 25 μM gefitinib+4 μM rofecoxib, 25 μM gefitinib+12 μMrofecoxib, 25 μM gefitinib+36 μMrofecoxib and 12 μM rofecoxib+12, 5 μM gefitinib. The survival of cells after treatment with drugs alone and in combination was compared to control performed with DMSO at the same final concentration of that present in medium with drugs. Values were reported as means ± SD of three independent experiments, and asterisks indicate significant difference (P < 0.05) of the treatment with two drugs vs single drug calculated by Student's t-test.

Cell growth of Ist-Mes-1, Ist-Mes-2 and MPP89 cells treated with four combinations of gefitinib-rofecoxib was determined by the cell proliferation kit. The concentration of drugs required to obtain a 25% inhibition (IC_25_) of proliferation in vitro, was used to test the effectiveness of the rofecoxib and gefitinib association in each of the cell lines. A beneficial effect of a simultaneous use of both drugs was not observed in Ist-Mes-1 and MPP89 cell lines (data not shown). The exposure to the two drugs induced an effect which was less severe than would be expected from the sum of the effects that each drug would produce on its own. One drug, therefore, counteracted some of the effects of the other. To verify whether this effect was reversed by a lower concentration of drugs, the concentrations of drugs tested on Ist-Mes-2 were also used in Ist-Mes-1 and MPP89. The dosage used for the cell lines was 25 μM of gefitinib with increasing doses of rofecoxib (4, 12 and 36 μM) and 12 μM rofecoxib with 25 μM gefitinib (Figure 4). None of the combinations produced any significant inhibition of cell proliferation with respect to the single drugs, except in Ist-Mes-2, where a synergistic effect of the two drugs was detected (Table 1). In particular, concentration of 12 μM rofecoxib+25 μM gefitinib led to a significant decrease in cell proliferation (21%) compared to 12 μM rofecoxib (45%) and 25 μM gefitinib (45%) alone (Figure 4). Otherwise, treatments with 25 μM gefitinib+4 μM rofecoxib and 25 μM gefitinib+36 μM rofecoxib caused a reduction of 25% and 19% respectively, compared to treatments with single drugs (45% for 25 μM gefitinib, 95% for 4 μM rofecoxib and 36% for 36 μM rofecoxib).

**Table 1:** Effect of gefitinib and rofecoxib combination in MM cell lines according to the Chou and Talalay method

Cell Lines	Schedule	FA	CI	Effect
Ist-Mes-2	12 μM R+12.5 μM G	0.59	0.74	moderately synergistic
	
	12 μM R+25 μM G	0.78	0.55	Synergistic
	
	25 μM G+4 μM R	0.75	0.48	Synergistic
	
	25 μM G+36 μM R	0.81	0.8	moderately synergistic

Ist-Mes-1	12 μM R+12.5 μM G	0.27	> 1.1	Antagonistic
	
	12 μM R+25 μM G	0.20	> 1.1	Antagonistic
	
	25 μM G+4 μM R	0.35	> 1.1	Antagonistic
	
	25 μM G+36 μM R	0.17	> 1.1	Antagonistic

MPP89	12 μM R+12.5 μM G	0.22	> 1.1	Antagonistic
	
	12 μM R+25 μM G	0.16	> 1.1	Antagonistic
	
	25 μM G+4 μM R	0.60	> 1.1	Antagonistic
	
	25 μM G+36 μM R	0.45	> 1.1	Antagonistic

Note: G indicates gefitinib, R indicates rofecoxib, FA denotes the fraction of growth affect of drug-treated cells compared with control cells and CI denotes the combination index.

### Effect of Rofecoxib and Gefitinib on p27, p21 and p-AKT expression

**Figure 5:**
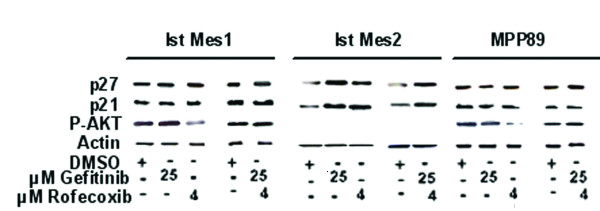
**Effect of gefitinib and rofecoxib on p21, p27 and p-AKT**. Ist-Mes-1, Ist-Mes-2 and MPP89 cell lines treated with gefitinib 25 μM , rofecoxib 4 μM and the combination gefitinib 25 μM +rofecoxib 4 μM for 48 hours were used to evaluate the effect of treatment on p21, p27, p-AKT and AKT in western blot. Actin was used as loading control. An increase in the amount of p27 and p21 in Ist-Mes-2 cells but no significant change in Ist-Mes-1 and MPP89 was reported. Phosphorylated AKT (p-AKT) was significantly reduced in Ist-Mes-1 and MPP89 by treatment with rofecoxib. No detectable p-AKT was found in Ist-Mes-2 cells.

To determine the biochemical mechanisms of drug-induced growth inhibition in MM, we evaluated the effect of gefitinib and rofecoxib on cell cycle inhibitors p21 and p27. According to the results obtained, illustrated in Figure 2, 4 and 5, we observed expression of p21 and p27 proteins only in Ist-Mes-2 cells (Figure 5). Indeed, in this cell line, treatment with single agent or rofecoxib and gefitinib as combination induced a significant increase of p21 and p27. Interestingly, in Ist-Mes-1 and MPP89 no differences in p21 and p27 were observed, when compared to control (Figure 5). Rofecoxib strongly inhibited constitutive p-AKT in Ist-Mes-1 and MPP89. Of note, gefitinib had no effect on baseline p-AKT in the Ist-Mes-1 and MPP89. In both cell lines MPP89 and Ist-Mes-1, association of the two drugs did not act on p-AKT, confirming an antagonistic effect of gefitinib in combination with rofecoxib. There was no detectable p-AKT despite abundant expression of AKT in Ist-Mes-2 cells. It is possible that in this cell line the AKT pathway may not be active, compared to Ist-Mes-1 and MPP89. Together, these data suggest that the response of MM cell lines upon gefitinib treatment is influenced by the activation of AKT. This would explain the lower sensitivity of Ist-Mes-1 and MPP89 cell lines with p-AKT.

## Discussion

We have demonstrated in the Ist-Mes-2 MM cell line a synergistic effect on the inhibition of cell growth between the active small molecule inhibitor of EGFR, gefitinib and rofecoxib, a drug that specifically targets COX-2,. Interestingly, the other two cell lines sensitive to treatment with single drugs, Ist-Mes-1 and MPP89, did not display this synergistic effect. As already described [30], COX-2 protein was appreciable in MPP89, Ist-Mes-2 and Ist-Mes-1. We demonstrated that EGFR phosphorylation was induced upon EGF treatment in over-expressing COX-2 MM cell lines and that, in these cell lines, EGFR inhibition with gefitinib and COX-2 inhibition with rofecoxib lead to decreases in proliferation. Gefitinib or rofecoxib treatment leads to primarily cytotoxic effects in Ist-Mes-1, Ist-Mes-2 and MPP89 cell lines. This is supported by the cytotoxicity observed in our cell proliferation assays. This is the first time that a cytotoxic effect has been observed on Ist-Mes-1, Ist-Mes-2 and MPP89 cell lines treated with gefitinib or rofecoxib. Previously, a study reported that gefitinib treatment leads primarily to cytostatic rather cytotoxic effect in MM cell lines [31]. As analyzed by Western blotting, there appears to be no significant differences in the amount of EGFR present in Ist-Mes-1 and Ist-Mes-2 cells, although the latter is much more sensitive to the effects of gefitinib (Figure 3). Thus, in MM cell lines, sensitivity to gefitinib inhibition is not strictly related to the amount of EGFR. In addition, in this study, to further substantiate the effect of gefitinib on the EGFR down-regulation pathway, we showed the inhibitory action of gefitinib on phosphorylation of the tyrosine kinase domain of the EGFR in MPP89, Ist-Mes-1 and, Ist-Mes-2 after treatment with EGF. In this context the increase of p-AKT was reversed by gefitinib in Ist-Mes-1 and MPP89, whereas no change of p-AKT in Ist-Mes-2 was observed, because levels of activated AKT were non-detectable. Gefitinib inhibition did not affect the basal p-AKT status in Ist-Mes-1 and MPP89. These data strongly confirm that EGF produced an increase of p-AKT in the less sensitive gefitinib cell lines. In accordance with these observations, the Ist-Mes-2 cell line, sensitive to gefitinib, was the only cell line in which activation of AKT failed in the presence of EGF. Indeed, it has been previously reported that persistent activity of the PI3K/Akt and/or Ras/Erk pathways is associated with gefitinib resistance of NSCLC cell lines [32]. PI3K/Akt signalling pathway is negatively regulated by the tumour suppressor gene phosphatase and tensin homologue (PTEN). Over-expression of PTEN engenders apoptosis in MM by AKT hypophosphorylation [33]. In light of these facts it is possible to suppose that over-expression of PTEN could be the basis of hypophosphorylated Akt in the Ist-Mes-2 cell line. Further investigations are required to better clarify this mechanism.

Interestingly, reduction of p-AKT was observed in Ist-Mes-1 and MPP89 treated with rofecoxib, suggesting this pathway is responsible for a reduction of cancer cell survival in these cell lines. In MPP89 and Ist-Mes-1 treatment with gefitinib and rofecoxib in combination was not effective. In these cell lines the effect of rofecoxib on the phosphorylation of AKT was counteracted by the addition of gefitinib. Only in Ist-Mes-2, the cell line where p-AKT was not detectable, did the combination of rofecoxib and gefitinib result in a synergistic effect. In order to obtain a better understanding of the growth-inhibitory effect of rofecoxib and gefitinib, we analyzed the expression of two cell cycle inhibitors, p21 and p27, in response to the in vitro treatment of cells with single drugs or in combination. It is well documented that inhibition of the EGFR dependent pathway induces a perturbation of cell cycle progression and notably G1 arrest [34]. p21 and p27 are able to arrest the growth of cells in the G1 phase of the cell cycle [35]. The growth inhibitory effect induced by gefitinib in Ist-Mes-2 increased expression of both p27 and p21. On the contrary, no variation in levels of p21 and p27 was observed in Ist-Mes-1 and MPP89. Indeed, p21 is critical for the activity of NSAID drugs and has been shown to play a role in MM progression [36]. Rofecoxib, alone and in combination with gefitinib, increased the expression of p21 and p27 only in Ist-Mes-2. Median effect analysis using the CI method of Chou and Talalay [29] confirmed a synergistic interaction between rofecoxib and gefitinib in Ist-Mes-2. In contrast, the combination of rofecoxib and gefitinib was not effective (antagonistic interaction) in Ist-Mes-1 and MPP89. These data, when considered together, indicate that gefitinib and rofecoxib alone and in combination are effective only in Ist-Mes-2, the cell line in which p27 and p21 are modulated and in which the active form of AKT was un-detectable. These results suggest that the differences in the susceptibility to drugs could be due to the differences in the signalling pathways affected, in addition to the responses that may depend on cell type. Further investigations will be undertaken to identify the mechanisms that underlie these differences in sensitivity of MM cell lines to single agents and their combinations, to identify new proteins involved in drug resistance. These proteins could subsequently be used as prognostic factors for drug resistance, thereby enabling prediction of response before starting treatment, in order to achieve a "tailored" therapy.

## Abbreviations

MM: malignant mesothelioma; COX: cyclooxygenase; COX-1: cyclooxygenase-1; COX-2: cyclooxygenase-2, PGE2: prostaglandin-E2; EGFR: epidermal growth factor receptor; GAPDH: glyceraldehyde-3-phosphate dehydrogenase; PCR: Polymerase Chain Reaction; p21: p21 CIP1/WAF1; p27: p27KIP1; ERK: extracellular signal-regulated kinase; PI3/AKT: phosphoinositide-3-kinase; NSAID: Non-Steroidal Anti-Inflammatory Drugs.

## Competing interests

The authors declare that they have no competing interests.

## Authors' contributions

DS carried out experiments, interpreted the results, CC carried out experiments interpreted the results, IC participated in the RT-PCR experiments, AV participated in the study coordination, AB assisted with the draft of the manuscript, AS critically revised the manuscript, RG designed the project, analyzed the results and wrote the manuscript. All authors read and approved the final manuscript.
